# Ghrelin and Leptin Concentrations in Patients after SARS-CoV2 Infection

**DOI:** 10.3390/jcm12103551

**Published:** 2023-05-18

**Authors:** Justyna Kuliczkowska-Płaksej, Aleksandra Jawiarczyk-Przybyłowska, Agnieszka Zembska, Katarzyna Kolačkov, Joanna Syrycka, Marcin Kałużny, Beata Polowczyk-Kawałko, Eliza Kubicka, Marek Bolanowski

**Affiliations:** Department and Clinic of Endocrinology, Diabetes and Isotope Therapy, Wrocław Medical University, Wybrzeże Pasteura 4, 50-367 Wroclaw, Poland

**Keywords:** COVID-19, ghrelin, leptin, infection

## Abstract

SARS-CoV2 infection can lead to severe cytokine storm especially in obese patients. Ghrelin acts not only as an appetite regulator but can also play a key role in the immune reaction. Leptin, secreted mainly by the white adipose tissue, can act as a pro-inflammatory cytokine. The crucial question is whether or not the cytokine storm in COVID-19 patients with obesity is linked to adipokine dysregulation. The aim of this study was to assess ghrelin and leptin concentrations in patients 6 months after SARS-CoV2 infection in comparison to a control group considering the influence of sex. The study group included 53 patients with a history of COVID-19 and 87 healthy subjects in the control group. Leptin and ghrelin concentrations as well as hormonal and biochemical parameters were measured. A significantly higher ghrelin concentration was observed in the COVID-19 group in comparison to the control group, with a statistically significant impact of sex on the relationship between COVID-19 and ghrelin concentration, which was lower in the males. No statistically significant differences in leptin concentration were observed between the groups. A significant negative correlation was observed between ghrelin and testosterone and morning cortisol levels in the COVID-19 group. The current study showed that ghrelin levels were significantly higher in patients 6 months after a mild course of SARS-CoV2 infection. To confirm the hypothetical protective role of ghrelin in the inflammatory process, it would be necessary to compare serum ghrelin levels between patients after mild and severe courses of COVID-19. Due to the small sample size and the lack of patients with a severe course of COVID-19, these observations need further investigation. There were no differences in leptin concentrations between the COVID-19 patients and the control group.

## 1. Introduction

SARS-CoV2 infection can provoke a cytokine storm due to the pro-inflammatory overproduction of chemokines and cytokines, which in turn leads to extensive multiorgan injury [1,2,3]. A cytokine storm leads to severe clinical manifestations or even acute mortality in critically ill patients with COVID-19. Impaired acquired immune responses and uncontrolled inflammatory innate responses may be the underlying mechanism of this complication [1,2,3,4,5]. Targeted control of the cytokine storm through immunomodulators and cytokine antagonists is essential to improve the survival rate of patients with COVID-19 [6,7]. There is a hypothesis that exogenous ghrelin in patients with COVID-19 could alleviate some of the severe symptoms that lead to cytokine storm syndrome, but so far, no such therapy has been attempted [7]. Ghrelin, a 28-amino-acid acylated peptide produced and secreted predominantly by the enteroendocrine cells of the stomach, is a potent inducer of food intake and also increases adiposity [8,9,10]. Ghrelin can act in the hypothalamus to regulate food intake, in the hippocampus to regulate neurogenesis, and in the olfactory bulb to regulate food-seeking behaviors [8,9]. Ghrelin-producing cells are present not only in the digestive system organs but also in many other tissues [11,12]. Despite the regulation of appetite, ghrelin influences many other biological actions, including having effects on glucose homeostasis, hormone secretion, and cell proliferation and survival and furthermore plays a key role in regulating immune function and inflammation [13,14,15,16,17,18,19]. Recent studies indicate that ghrelin has protective effects in acute lung injury models [10,20,21,22,23,24]. According to previous reports, treatment with ghrelin improved morphologic damage and pulmonary parameters and decreased serum pro-inflammatory cytokine levels in a pancreatitis-induced acute lung injury model [6]. There is only one publication on ghrelin levels in COVID-19 patients, but it concerns only the effect of ghrelin on appetite [25].

Leptin is a hormone mainly secreted by the white adipose tissue. Its secretion is a satiety signal, and its levels rise with increasing fat mass. Through its central function, leptin affects food intake, physical exercise, energy balance, and adipose tissue mass [26,27,28]. Nonetheless, leptin is also thought to be a pro-inflammatory cytokine, and it also shares structural similarities with inflammatory cytokines, such as IL-6, IL-12, and granulocyte-colony-stimulating factors [29,30,31,32,33,34]. High circulating leptin might involve the dysregulation of pro-inflammatory cytokine in obesity. The crucial question is whether or not the cytokine storm in COVID-19 patients with obesity is linked to leptin dysregulation.

The study aimed to assess the occurrence of differences in the concentration of ghrelin and leptin between patients 6 months after SARS-CoV2 infection and a control group as well as selected biochemical, hormonal, anthropometric, and densitometric parameters, considering the influence of sex. The results of our study could shed more light on the interplay of various hormonal and immune factors during SARS-CoV2 infection. Despite many animal studies supporting the anti-inflammatory potential of ghrelin, there are only a few studies concerning ghrelin use in inflammatory disorders in humans. 

## 2. Materials and Methods

The study group included 53 patients with a history of COVID-19 infection (38 females and 15 males, aged 45.33 ± 12.16), and 87 healthy subjects were included in the control group (50 females and 37 males, aged 46.55 ± 12.39).

The inclusion criterion for the study group was a history of COVID-19 infection (up to 6 months from the onset of the disease) not requiring hospitalization. The exclusion criteria were any chronic, inflammatory diseases in their past medical history, diabetes, nicotinism, alcohol abuse, and glucocorticoid use. In the control group, we included patients without COVID-19 infection in their medical history, with negative antibodies indicating a history of SARS-CoV2 infection. All of the participants were recruited from the Department of Endocrinology, Diabetes and Isotope Therapy, Wroclaw Medical University. The bioethics committee of Wroclaw Medical University approved the protocol of the study. All subjects signed informed consent forms in line with the Declaration of Helsinki.

The patients’ weight (kg) and height (m) were recorded, and their body mass index (BMI) was calculated. Fasting venous blood samples were collected from all of the participants.

Homeostasis model assessment of IR index (HOMA-IR index) is calculated by the formula HOMA-IR = [glucose (mmol/L) × insulin (µU/L)]/22.5. The quantitative insulin sensitivity check index (QUICKI index) is calculated by using QUICKI index = 1/[Log Insulin (µU/mL) + Log Glucose (mg/dL)]. The FIRI (fasting insulin resistance index) is estimated as the product of fasting plasma glucose and fasting plasma insulin divided by 25 (FIRI = (glucose × insulin/25). Estimates of the glomerular filtration rate (eGFR) are based on creatinine and patient characteristics. To calculate the results, we use the following calculation formula: eGFR = 175 × [creatinine × 0.011312]^−1.154^ × [age]^−0.203^ × [0.742 if female].

The dual-energy X-ray absorptiometry (DXA) method (Horizon A Hologic densitometer) was used to assess the bone mineral density (BMD) of the lumbar spine (L1–L4) and the femoral neck. The results were expressed in absolute values (grams per square centimeter) as well as the SD from the peak bone mass (T-score) and the expected mass for the age-matched population (Z-score).

We used TBS iNsight software (v 3.0.3.0, Med-Imaps, Pessac, France) to analyze all of the TBS values, using lumbar spine BMD DXA files from the database. The TBS exams were performed by the same person.

### 2.1. Leptin and Ghrelin

The plasma samples were obtained by centrifugation of the whole blood, aliquoted, and stored at −80 °C until use. Leptin as well as ghrelin (total) level concentrations were evaluated by radioimmunoassay (RIA) using EMD Millipore Corporation kits (EMD Millipore Corporation, Billerica, MA, USA) following the manufacturer’s instructions. Both assays proceeded by utilizing radioactive ^125^I-labeled forms of the antigen of human leptin and ghrelin, and their specific antiserum.

The concentration of the samples was determined by interpolation of the standard curve and measured using a gamma counter (Wizard 1470, Perkin Elmer, Waltham, MA, USA). The sensitivity level of this assay was 0.437 ng/mL for leptin and 93.0 pg/mL for ghrelin.

### 2.2. Statistical Analysis

The statistical analysis was performed using R for Windows statistical software (version 4.0.4, Vienna, Austria). The variables were presented as the mean ± standard deviation. The Shapiro–Wilk test was used to check the normality of the data. The variables were not normally distributed; hence, we opted for non-parametric tests when appropriate. The Mann–Whitney test (for two groups) or the Kruskal–Wallis test (for more than two groups) were applied to compare the quantitative variables. The categorical variables were compared by the chi-square test or Fisher’s exact test. Correlations between parameters were calculated using Spearman’s rank correlation test. *P*-values < 0.05 were considered significant.

## 3. Results

The general characteristics of the study group and the control group are presented in Table 1. 

There were no statistically significant differences in the anthropometric parameters between the groups, as well as between the females and males.

A statistically higher ghrelin concentration was observed in the COVID-19 group in comparison to the control group (p = 0.003) (Table 1). In addition, we revealed a statistically significant impact of sex on the relationship between COVID-19 and ghrelin concentration, which was statistically significantly lower in the males (p = 0.020). We did not observe any difference in ghrelin levels in men regardless of the study group. Serum ghrelin was significantly higher in the females from the COVID-19 group in comparison to the females from the control group (Table 2). 

There were no statistically significant differences in the leptin concentrations between the study group and the control group, but we observed statistically significant lower concentrations of leptin in males in both of the analyzed groups (*p* = 0.033).

Both groups did not vary in their serum creatinine level (*p* = 0.275), but we observed a statistically significant disparity in the eGFR (estimated glomerular filtration rate) (*p* = 0.04). In addition, sex and COVID-19 infection were independent factors affecting the observed difference (*p* = 0.038, *p* = 0.055, respectively).

We did not observe significant differences in total cholesterol, LDL cholesterol, HDL cholesterol, and triglyceride levels in either of the groups. There were also no differences in C-reactive protein, glucose, and insulin levels, as well as insulin resistance index values (HOMA-IR, QUICKI, FIRI) in either group (Table 1).

**Figure 1:**
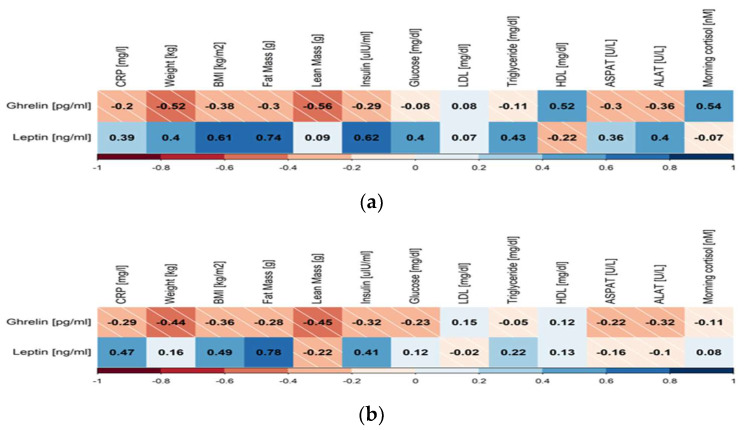
Heat map of correlations between ghrelin and leptin and the anthropometrics, biochemical, and hormonal parameters in the study group (**a**) and the control group (**b**).

In the study group, we observed a significant correlation between leptin concentration and metabolic parameters as well as CRP levels in both groups. In addition, in the COVID-19 group, we observed a statistically significant negative correlation between ghrelin and testosterone as well as ghrelin and morning cortisol levels. In the control group, the same correlation was observed only for testosterone (Figure 1, Table S1). We also observed a negative correlation between ghrelin and BMI, weight, and lean mass in the COVID-19 group as well as in the control group.

## 4. Discussion

The understanding of the actions and pathogenicity of SARS-CoV2 has become the center of interest especially due to the observation that a severe course of COVID-19 was observed in some specific patient populations [35,36,37,38]. The reason for the worse course of SARS-CoV2 infection is the cytokine storm characterized by an overreaction of the immune system leading to multiple organ failure. It is yet not known what triggers a hyperinflammatory response in a certain number of patients; the search for factors contributing to such a course of infection has been ongoing. Cytokine storms significantly more often affected obese patients, mainly males, especially those suffering from cardiovascular disease and diabetes [39,40,41,42,43,44].

In our study, we analyzed a group of middle-aged patients who were slightly overweight, up to 6 months after the onset of mild SARS-CoV2 infection who did not require hospitalization. This group was compared with a group of healthy patients matched according to age and BMI. There were no significant differences in hormonal and metabolic parameters between both groups of patients. There was also no significant effect of sex on the association of COVID-19 with these parameters. However, a significantly higher concentration of ghrelin was observed in the group of patients with COVID-19 compared to healthy people of the same age and comparable BMI. Increased ghrelin levels in the course of COVID-19 may indicate a relationship between ghrelin and the immune system. Ghrelin, aside from its known role as an appetite- and energy-expenditure-regulating agent, has been shown to possess specific properties that inhibit cell proliferation, oxidative stress, inflammation, and apoptosis [11,12,16,45,46]. Ghrelin and its receptors (GHS-R) have been detected in lung tissue, indicating that the peptide may play an important role in respiratory system regulation [11,12]. According to some authors, ghrelin is also able to decrease basal and tumor necrosis factor α (TNF-α)-induced chemotactic cytokine production and mononuclear cell adhesion in human vascular endothelial cells [47,48]. Furthermore, ghrelin and GHS-R are expressed in human monocytes and T lymphocytes and activation of GHS-R subsides the expression of pro-inflammatory cytokines such as IL-6, IL-1β, and TNF-α [16,17,18,19,45,49]. Only a few human trials have been conducted on the use of ghrelin in inflammatory disorders and all of them demonstrated a reduction of inflammation during the treatment with ghrelin. Moreover, ghrelin interacts with receptors involved in SARS-CoV2 entry such as ACE2, which suggests that it has antiviral activity. There is only one study on the level of ghrelin in patients with COVID-19 concerning its influence on appetite in the context of SARS-CoV2 infection. The authors showed no significant changes in the level of serum ghrelin in COVID-19 patients, but they hypothesize that it might be possible that the ghrelin showed potential changes in the saliva compared to the effect in the blood. Unfortunately, there is no detailed information about the course of SARS-CoV2 infection or the inflammatory parameters in the study group in this publication. There is also no information about the gender differences in serum ghrelin levels. The authors only stated that there were more men in the COVID-19 group [25].

Although there is an absence of obvious clinical evidence on the therapeutic effects of ghrelin on COVID-19 and the associated complications, preclinical studies have demonstrated that ghrelin is able to ameliorate the severity of acute lung injury by reducing lung fluid accumulation, hypoxemia, and cytokine secretion, all of which also occur in COVID-19-associated acute respiratory distress syndrome [50,51]. In our study, we observed a negative correlation between ghrelin and body mass, and BMI. The same correlations were demonstrated in the healthy control group. In humans, several studies have demonstrated that ghrelin levels are negatively correlated with body mass and could be used as a supportive treatment for cachexia or anorexia nervosa [52,53].

We also observed a negative correlation between ghrelin and testosterone levels. Ghrelin is expressed in human testes, and testosterone supplementation suppresses total ghrelin in non-obese, eugonadal men. On the other side, in overweight or obese men with low testosterone levels, testosterone supplementation increased ghrelin during energy balance or had no effect during energy deficit [54,55,56,57]. Probably, the impact of testosterone on ghrelin is multifactorial, depending on the basal hormonal status, energy balance, and body mass. In addition, we revealed a statistically significant impact of sex on the relationship between COVID-19 and ghrelin concentration, which was statistically significantly lower in males. Therefore, we can hypothesize that the male gender could have a possible negative impact on the course of COVID-19 partly also by affecting ghrelin concentration. There are only a few publications concerning gender differences in plasma ghrelin, and the results of these studies have been contradictory. Some authors reported higher ghrelin concentrations in females whereas others did not observe any sex differences [58,59,60,61,62,63,64]. Makovey et al. reported higher plasma ghrelin in females and after adjustment for gender, fat mass, and body size, age was a significant predictor of plasma ghrelin levels [58]. In the older age group, plasma ghrelin levels were inversely associated with fat mass measures—in women, they were correlated inversely with BMI, total fat mass, and the fat mass/lean mass ratio, in comparison to men with abdominal fat mass [58]. In our study, serum ghrelin levels were significantly higher in women not only in comparison to men but also in women with COVID-19 in comparison to healthy women from the control group (Table 2).

We might only hypothesize that the existence of a significant difference in ghrelin levels between both groups in our study, despite the absence of obesity or other risk factors for a severe course of the disease, might indicate that during COVID-19 infection, increased ghrelin may contribute, among other factors, to the mild course of the infection, especially in women. It is also possible that ghrelin may play a role in the recovery process after SARS-CoV2 infection. To confirm these hypotheses, it would be necessary to compare serum ghrelin levels between the patients after a mild and severe course of COVID-19.

An Important factor that contributes to immune imbalance and worse prognosis in obese COVID-19 patients is chronically increased leptin. Leptin, as an inflammatory cytokine produced by visceral adipose tissue, could amplify systemic inflammation in COVID-19 patients with obesity [28,33,34,65,66]. It has structural homology with cytokines, such as IL-6, IL-11, and IL-12, and has an impact on the stimulation and proliferation of various immune cells including monocytes, natural killer cells, and T helper cells [29,30,67,68,69,70]. The crucial question is whether or not the cytokine storm in COVID-19 patients is linked to leptin dysregulation. Several studies have linked increased leptin with a worse course of SARS-CoV2 infection [34,49,71]. A large, observational study on healthy patients and patients with COVID-19 divided into two groups according to COVID-19 severity (moderate and severe) showed a reduced adiponectin to leptin ratio which is a marker of adipose tissue dysfunction. Patients with cardiometabolic disturbances had the lowest ratio which might contribute to worse outcomes in COVID-19 [72]. In a study on 31 COVID-19 patients, the serum leptin levels of mechanically ventilated patients were significantly higher in comparison with the control group [71]. Wang et al. observed that patients with COVID-19 with a high BMI had significantly high levels of leptin, which were associated with inflammatory mediators and disease severity in such patients. Of note, leptin levels were increased in patients with COVID-19 compared with the controls as well as in severe patients with COVID-19 compared with mild patients [34]. In our study, we did not observe significant differences in leptin levels between COVID-19 and healthy patients probably due to only slightly elevated body mass and BMI values in both groups. Moreover, the COVID-19 patients and the control group were matched by BMI. We demonstrated the significant positive correlation of leptin with body mass, BMI, fat mass, insulin, HOMA value, LDL cholesterol, triglycerides, and alanine transaminase, which potentially link leptin to an adverse metabolic profile. Taking into account only slightly elevated BMI values and a mild course of SARS-CoV2 infection in our COVID-19 group, it seems that leptin levels in such populations of patients have no significant impact on COVID-19 outcomes.

This study focused on the interpretation of ghrelin and leptin concentrations 6 months after COVID-19 infection in patients without obesity, with a mild course of the disease. To our best knowledge, it is the second study concerning ghrelin levels in patients with SARS-CoV2 infection. Due to the small sample size, which is the major limitation of our study, the results are preliminary and need further investigation. The second limitation of the work is the lack of assessment of ghrelin concentration in patients with severe forms of COVID-19. According to the obtained results, the higher levels of ghrelin in patients who had the mild form of SARS-CoV2 infection for up to six months previous in comparison to the healthy group might indicate the possible role of ghrelin in the regulation of the immune process. Since ghrelin represses inflammation by reducing oxidative stress and cytokine production, it might play a similar role in protecting against lung injury associated with COVID-19. In subjects without obesity and concomitant disorders, the good functioning of ghrelin could possibly block virus–cell interaction, preventing cytokine storm and multi-organ injury. It has been suggested that leptin could be involved in the etiology of several effects commonly observed in patients with COVID-19 and obesity. In our study, the leptin levels did not differ between both groups of patients, probably because the patients had only a slightly increased BMI. Anyway, it is important to note that we observed a significant correlation between leptin and metabolic parameters as well as CRP levels. We could suppose that leptin might be involved in the dysregulation of pro-inflammatory cytokines in obesity, which is the leading cause of high morbidity and mortality in patients with SARS-CoV-2 infection.

It would be reasonable to determine the level of ghrelin and leptin in patients with different courses of SARS-CoV2 infection (mild and severe), which would probably shed more light on the role of ghrelin and leptin in the immune response in COVID-19 patients.

## Figures and Tables

**Table 1:** The general characteristics of the study group and the control group.

	Study Group (*n* = 53)			Controls (*n* = 87)			
	Mean ± SD	Median	IQR	Mean ± SD	Median	IQR	*p-*Value
Age (years)	45.339 ± 12.160	44	21.5	46.556± 12.391	44	18.5	0.53
Height (m)	1.689 ± 0.078	1.665	0.120	1.689 ± 0.097	1.690	0.147	0.918
Weight (kg)	74.604 ± 16.608	76	23	77.351 ± 16.531	76	22	0.318
Fat (%)	32.521 ± 6.705	31.5	9.5	32.847 ± 6.695	33.2	11.2	0.793
Fat mass (g)	24,559.95 ± 7821.173	22,862.75	14,144.92	25,903.17 ± 7752.641	24,422.5	10,899.90	0.232
Lean mass (g)	48,173.30 ± 11,654.69	45,910.9	17,411.65	50,480.01 ± 11,916.18	49,288.0	18,756.40	0.253
Ghrelin (pg/mL)	1326.095± 848.215	1190.555	579.812	1001.010 ± 424.116	901.390	44.590	0.003
Leptin (ng/mL)	26.601 ± 23.290	18.475	29.847	27.166 ± 25.627	19.475	25.910	0.852
ALAT (U/L)	26.3 ± 18.43	20.5	14.75	25.765 ± 17.109	20	16	0.888
ASPAT (U/L)	22.325 ± 8.319	19.5	8.75	20.921 ± 7.42	19	8	0.532
Total cholesterol (mg/dL)	191.582 ± 39.863	189	57	195.307 ± 40.435	195.5	64.5	0.455
LDL cholesterol (mg/dL)	112.800 ± 33.386	109	49	118.705 ± 33.509	118.5	46.25	0.231
HDL cholesterol	58.473 ± 14.961	57	23	55.216 ± 12.399	55	20	0.336
Triglyceride (mg/dL)	100.8 ± 62.399	86	73	107.216 ± 66.302	92	65.75	0.495
CRP (mg/L)	1.544 ± 1.302	1.2	1.325	1.875 ± 2.391	0.9	1.3	0.0776
Glucose	87.779 ± 9.386	86	14	92.209 ± 15.598	88	13	0.143
Insulin (μIU/mL)	9.652 ± 8.923	6.9	7.215	10.133 ± 8.406	7.86	7.485	0.538
HOMA-IR	2.253 ± 2.249	1.578	1.798	2.377 ± 2.201	1.748	2.09	0.528
QUICKI	0.364 ± 0.049	0.356	0.062	0.360 ± 0.049	0.351	0.064	0.528
FIRI	2.027 ± 2.024	1.420	1.618	2.129 ± 1.981	1.573	1.888	0.528
Testosterone (ng/mL)	1.255 ± 1.728	0.270	2.122	1.719 ± 1.932	0.349	3.180	0.308
eGFR (mL/min/1.73 m^2)^	88.057 ± 14.059	88	18.5	94.012 ± 14.198	93	19	0.044

Significant results (*p* < 0.05).

**Table 2:** Post hoc comparisons of ghrelin—sex and group.

Comparison				Difference	SE	t	df	*p*-Value
Sex	Group	Sex	Group					
Female	COVID	Female	CONTROL	446.0	125	3.569	142	0.002 *
Female	COVID	Male	COVID	711.6	170	4.186	142	<0.001 *
Female	COVID	Male	CONTROL	660.0	132	5.015	142	<0.001 *
Female	CONTROL	Male	CONTROL	214.0	124	1.725	142	0.260
Male	COVID	Female	CONTROL	−265.6	164	−1.618	142	0.260
Male	COVID	Male	CONTROL	−51.6	169	−0.305	142	0.761
Sex	Group	Number	Median	IGR	Mean ± SD			
Female	COVID	39	1289.29	620.64	1542.10 ± 912.36			
Female	CONTROL	50	998.93	490.158	1096 ± 482.52			
Male	COVID	17	801.44	552.38	830.54 ± 348.02			
Male	CONTROL	40	847.68	305.49	882.10 ± 303.07			

* Significant results (*p* < 0.05).

## Data Availability

The original contributions presented in the study are included in the article; further inquiries can be directed to the corresponding authors.

## Supplementary Materials

The following supporting information can be downloaded at: https://www.mdpi.com/article/10.3390/jcm12103551/s1. Table S1: Correlation of Ghrelin and Leptin with anthropometrics, biochemical and hormonal parameters.
