# Supplementary material for: Ghrelin and Leptin Concentrations in Patients after SARS-CoV2 Infection
Source: J Clin Med. 2023 May 18;12(10):3551. doi: 10.3390/jcm12103551 (PMC10218943; doi:10.3390/jcm12103551)
Supplement: Supplementary file 1 [file jcm-12-03551-s001.zip › jcm-2186316-supplementary.pdf]

Table S1. Correlation of Ghrelin and Leptin with anthropometrics, biochemical and hormonal parameters.

	**Study group (n = 53) Ghrelin (pg/ml)**		**Controls (n = 87) Ghrelin (pg/ml)**			**Leptin (ng/ml) Study group**		**Leptin (ng/ml) Control group**	
	**R**	**P**	**R**	**P**		**r**	**P**	**r**	**P**
CRP (mg/dl)	-0.2	0.33	-0.29	**0.028**	CRP (mg/dl)	0.39	**0.025**	0.47	**0.000**
Weight (kg)	-0.52	**0.0005**	-0.44	**0.000**	Weight (kg)	0.4	**0.015**	0.16	0.34
BMI (m^2/kg)	-0.38	**0.022**	-0.36	**0.006**	BMI (m2/kg)	0.61	**<0.001**	0.49	**<0.000**
Fat mass (g)	-0.30	0.95	-0.28	**0.039**	Fat mass (g)	0.7	**<0.0001**	0.78	**<0.000**
Lean Mass (g)	-0.56	**0.0003**	-0.45	**0.0006**	Insulin (uIU/ml)	0.62	**< 0.001**	0.41	0.0013
Insulin (µIU/ml)	-0.29	0.11	-0.32	**0.018**	Glucose (mg/dl)	0.4	**0.015**	0.12	**0.48**
Glucose (mg/dl)	-0.08	0.76	-0.23	0.15	Triglyceride (mg/dl)	0.43	0.076	0.22	0.13
LDL cholesterol (mg/dl)	0.08	0.76	0.15	0.48	HOMA/FIRI	0.6	**<0.0001**	0.37	**0.04**
Triglyceride (mg/dl)	-0.11	0.64	-0.04	0.8	Quicki	- 0.6	**< 0.0001**	-0.37	0.04
HDL (mg/dl)	0.52	**0.0004**	0.12	0.5	LH (mIU/ml)	0.4	**0.012**	0.34	**0.096**
Morning Cortisol (nM)	0.54	**0.012**	-0.11	0.63	FSH (mIU/ml)	0.45	**0.005**	0.35	**0.006**
Testosterone (ng/ml)	- 0.53	**0.0036**	- 0.3	**0.021**	Testosterone (ng/ml)	- 2.9	0.09	-0.40	**0.0013**
Aspartate aminotransaminase (U/L)	-0.3	0.18	-0.22	0.23	Aspartate aminotransaminase (U/L)	0.36	0.9	-0.16	0.39
Alanine Aminotransaminase (U/L)	-0.36	0.083	-0.32	**0.019**	Alanine Aminotransaminase (U/L)	0.4	**0.04**	-0.10	0.57

significant results ($p<0.05$).
